# Identification of proteoforms of short open reading frame-encoded peptides in *Blautia producta* under different cultivation conditions

**DOI:** 10.1128/spectrum.02528-23

**Published:** 2023-10-02

**Authors:** Jerome Genth, Kathrin Schäfer, Liam Cassidy, Simon Graspeuntner, Jan Rupp, Andreas Tholey

**Affiliations:** 1 Systematic Proteome Research & Bioanalytics, Institute for Experimental Medicine, Christian-Albrechts-Universität zu Kiel, Kiel, Germany; 2 Department of Infectious Diseases and Microbiology, University of Lübeck, Lübeck, Germany; 3 German Center for Infection Research (DZIF), Partner Site Hamburg-Lübeck-Borstel-Riems, Lübeck, Germany; University of Minnesota, Minneapolis, Minnesota, USA

**Keywords:** gut microbiome, microproteins, short open reading frames, small open reading frames, sORF-encoded peptides, proteoforms, top-down proteomics

## Abstract

Short open reading frames (sORF) have the potential to encode small proteins, sORF-encoded peptides (SEP), which are less than or equal to 100 amino acids in length. These SEP have been associated with various physiological processes and are known for their potential roles in modulating immune responses, signaling pathways, and cellular functions. Using bottom-up and top-down proteomics, we identified 45 novel and formerly reported SEP under different growth conditions and stress exposure in single cultures of *Blautia producta* that plays a central role in mediating colonization resistance and dampening inflammation in the human gut microbiome. Top-down analysis improved identification confidence and allowed the detection of a number of full-length and N- or C-terminal truncated proteoforms of the SEP and of proteoforms carrying post-translational modifications such as disulfides, which provides hints for a wide functional potential of these molecules. The identification of the SEP in single cultures of this bacterium shows that, in contrast to previous results, the biosynthesis of specific SEP is not restricted to direct bacterial interactions within the microbiome but is dependent on growth and environmental conditions during cultivation.

**IMPORTANCE:**

The identification of short open reading frame-encoded peptides (SEP) and different proteoforms in single cultures of gut microbes offers new insights into a largely neglected part of the microbial proteome landscape. This is of particular importance as SEP provide various predicted functions, such as acting as antimicrobial peptides, maintaining cell homeostasis under stress conditions, or even contributing to the virulence pattern. They are, thus, taking a poorly understood role in structure and function of microbial networks in the human body. A better understanding of SEP in the context of human health requires a precise understanding of the abundance of SEP both in commensal microbes as well as pathogens. For the gut beneficial *B. producta*, we demonstrate the importance of specific environmental conditions for biosynthesis of SEP expanding previous findings about their role in microbial interactions.

## INTRODUCTION

Advancements in genome sequencing and open reading frame (ORF) prediction algorithms have led to an increasing number of predicted and assigned short ORF (sORF) in many bacterial genomes. Their gene products, short peptides or proteins less than 50–100 amino acids in length, have been referred to as microproteins, a common classification for those with functional domains (1), or as sProteins (2, 3) or sORF-encoded peptides (SEP; 4 – 6). SEP exhibit a wide range of essential biological functions and have been, for example, associated with glucose uptake, cell division, peptidoglycan synthesis, stress responses, virulence, and sporulation or acting as chaperones (7 – 10). Several studies have indicated or confirmed an anti-bacterial activity of SEP, emphasizing a potential involvement in bacterial-bacterial and bacterial-host interactions (3, 11, 12).

The discovery of SEP in the human gut microbiome has been hampered mainly due to the challenges in the correct genome annotation of sORFs and the applied proteomics technologies. With an increasing number of sequenced genomes and improved ORF prediction algorithms, thousands of highly conserved sORF have been discovered across various bacteria in the human microbiome, highlighting the vastly underestimated complexity of bacterial genomic and proteomic architecture (13). To address this challenge, databases derived from *in silico* six-frame translations of the whole-genome sequence (14, 15) or “integrated proteogenomics search databases” (iPtgxDB) have been developed (16). Compared with conventional six-frame translations, the iPtgxDB has consolidated annotations and predictions from different sources, considered alternative start sites, minimized redundancy, and covered the entire protein-coding potential of a prokaryotic genome (16).

Mass spectrometry (MS)-based proteomics techniques are essential to prove the translation and thus the existence of SEP within the proteome. However, their intrinsic small size and, in some cases, also their low abundance are potential factors that hamper their detection by the most widely employed bottom-up proteomics (BUP) approaches (4, 17, 18). In contrast, top-down proteomics (TDP) allows for direct analysis at the intact protein level, enabling the detection of co- and post-translational modifications (PTMs) and proteolytic processing (19), finally leading to the identification of proteoforms of SEP (4, 20).

SEP within the human gut microbiome received increasing attention in recent years. In a pioneering study (3), Petruschke and colleagues employed different protein extraction approaches in order to identify novel small proteins using a simplified model system of the human intestine microbiome (SIHUMIx; 21). The authors identified several novel SEP that were exclusively produced within the SIHUMIx community, but not in single isolated bacterial cultures, including previously undescribed SEP (BP3, BP5, BP8, BP11, and BP12) from *B. producta*, suggesting their potential functional role within the microbial community. However, the authors also noted the influence of varying growth and cultivation conditions on the identification of these proteins, indicating the need for further research to fully understand their function and relevance in the microbiome.

In the present study, which was motivated by our observation of fragments of several of these SEP in single cultures of *B. producta*, we aimed to identify optimized conditions for the biosynthesis of the reported and not yet identified SEP in *B. producta*. Employing both BUP and TDP approaches, together with strict criteria for the evaluation of the quality of the corresponding MS/MS spectra, we were able to confirm the presence of a number of the formerly reported SEP independent of the presence of other microorganisms, alongside with a number of novel SEP of unknown function.

## RESULTS AND DISCUSSION

### Workflow overview for SEP identification

**Fig 1:**
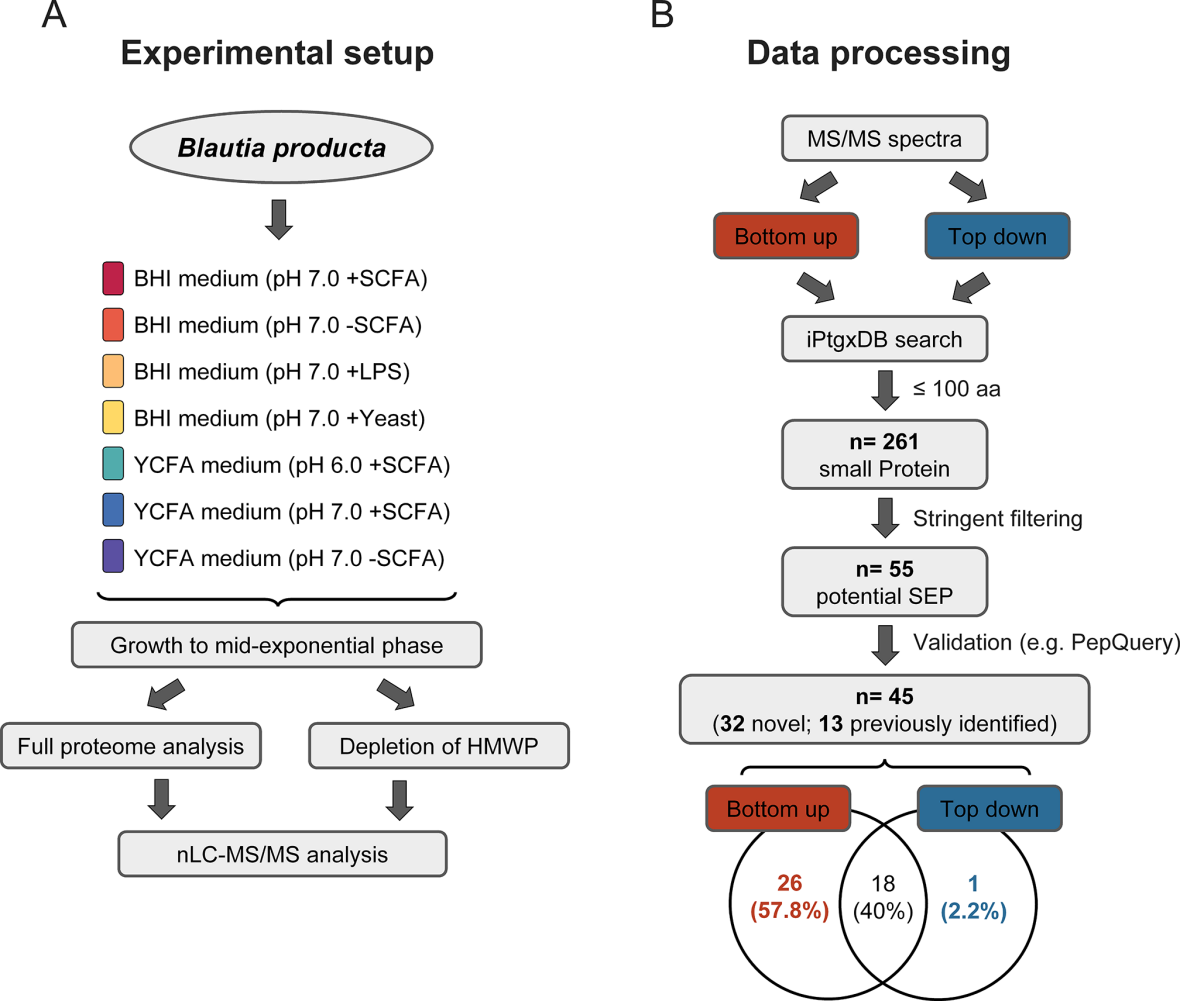
Workflow for the identification of SEP. (**A**) Overview of the experimental setup, which involves the cultivation of *B. producta* under seven different culture conditions, represented by different colors, followed by lysis of the bacterial cells using a freeze-thaw method. This was followed by a full-proteome analysis via bottom-up proteomics and the analysis of the low-molecular weight fractions after depletion of high-molecular weight proteins, by both bottom-up and top-down proteomics. (**B**) Proteogenomic workflow, including the use of both bottom-up and top-down MS/MS spectra that were searched against the iPtgxDB to identify novel SEP. After applying a size filter of smaller than 100 amino acids and implementing a stringent filtering regimen, a total of 45 SEP were identified.

*Blautia producta* was cultivated under seven conditions, with different combinations of culture media (Brain Heart Infusion [BHI] and Yeast extract, Casitone, and Fatty Acid [YCFA]), pH conditions, and supplements (yeast extract, short-chain fatty acids [SCFAs] or lipopolysaccharides [LPS]; Fig. 1A; Fig S1). After sampling at the mid-exponential phase and cell disruption, aliquots of these samples were subjected to full-proteome BUP analysis. In the remaining aliquot, high-molecular weight proteins were depleted using an optimized depletion protocol consisting of two precipitation steps to first remove lipids, cell membrane components, and metabolites and second the majority of high-molecular weight proteins with masses above *ca*. 15 kDa (22) (Fig. 1A). The remaining low-molecular weight (LMW) fraction was then analyzed both by a BUP and, for two growth conditions, additionally by an TDP analysis. To identify the potential SEP, the iPtgxDB was utilized (Fig. 1B) (16). To improve the reliability and accuracy of the peptide identification and minimize the risk of false-positive or false-negative results, respectively, a stringent filtering protocol for both BUP and TDP identifications was implemented that considered both the number of peptide (PSM, BUP-approach) and the proteoform (PrSM, TDP-approach) spectra matches and the quality of the MS/MS spectra (Fig. 1B). Both approaches, required at least three PSMs/PrSMs for Prodigal and ChemGenome predictions and least four PSMs/PrSMs for *in silico* ORF predictions (23). For TDP proteoform identification, a minimum C-Score of 40 (24), an *E*-value smaller than 1 × 10–5, and at least 10 matching fragment ions with a mass error less than 10 ppm were required (25, 26). The BUP filtering process for SEP included manual inspection of all assigned peptides (27) and required an MS^2^ spectrum to have a sequence tag of at least five consecutive b- or y-ions (28). Furthermore, putative non-canonical peptides were subjected to a two-step verification process: first using PepQuery (29) to assess possible sequence matches with hypothetical post-translational modifications and amino acid substitutions, and then, non-matched peptides were evaluated against the *B. producta* proteome using NCBI Protein Blast. This step was implemented to exclude peptides that exhibited a single amino acid variation when compared with a known protein sequence, as these variations may be the result of single-nucleotide polymorphisms and are more likely to be false positives.

Additionally, an *in silico* digest of the proteomes *of Saccharomyces cerevisiae*, pig, and cattle was performed to minimize the risk of false positives from protein carryover in the yeast extract or Brain Heart Infusion media and compared with the iPtgxDB digest. Only 119 out of 2 million peptides resulting from the *in silico* digestion of *S. cerevisiae* overlapped with iPtgxDB (Fig S2A), with the porcine and bovine proteomes showing a slightly higher peptide overlap Fig. S2 C through E. Treating leucine and isoleucine as equivalent in peptide sequence analysis led to the identification of additional peptides (Fig. S2B, D and F), including the peptide “RLLQDLK” and “EKEILEK” as part of BP7 and BP17, respectively. However, these findings did not lead to the exclusion of these SEP, as several other unique peptides of these were identified. These results suggest that the peptide identifications in this study are reliable and accurate, as potential contamination from the use of protein-containing yeast extract or brain heart infusion can be largely excluded.

Among the 45 SEP identified in this study, 14 of the 15 previously described *B. producta* SEP (BP1-BP14) (3) were further validated, while the remaining 31 SEP (BP16–BP46) are, to the best of our knowledge, reported by us for the first time at the protein level. Notably, one SEP (BP46) was only identified through top-down analysis.

#### Novel SEP identified by bottom-up proteomics

In the initial BUP analysis of the non-depleted proteome and the LMW fractions, a total of 2,379 proteins were identified across all seven cultivation conditions. After applying a size filter of ≤100 amino acids, 261 proteins (9.1%) were classified as small proteins. From these, 55 met the filtering criteria and underwent the two-step verification process as potential novel SEP, represented by 142 non-canonical peptides. Applying the stringent filtering, 35 peptides (24.6%) were excluded by PepQuery, of which 4 were disregarded based on their presence in the reference database, 12 did not confidently match with the MS/MS spectra, and 19 were either better matched to canonical peptides, random peptides, or better matched to reference peptides carrying potential post-translational modifications. The remaining 107 peptides passed the statistical threshold (*P* < 0.01) for matching the non-canonical sequence. BLASTp analyses led to the exclusion of an additional peptide and its corresponding SEP due to a single amino acid variation in comparison with a known protein sequence. This resulted in a total of 44 SEP identified via BUP (Fig. 1B; Table S2), of which 14 had been previously described (3). It is important to acknowledge single-peptide SEP identifications potentially suffer from higher false discovery rates (30). However, within this analysis, only 17 of the 44 SEP fell into this category with an average of 69 PSMs identified per peptide (Table S2).

**Fig 2:**
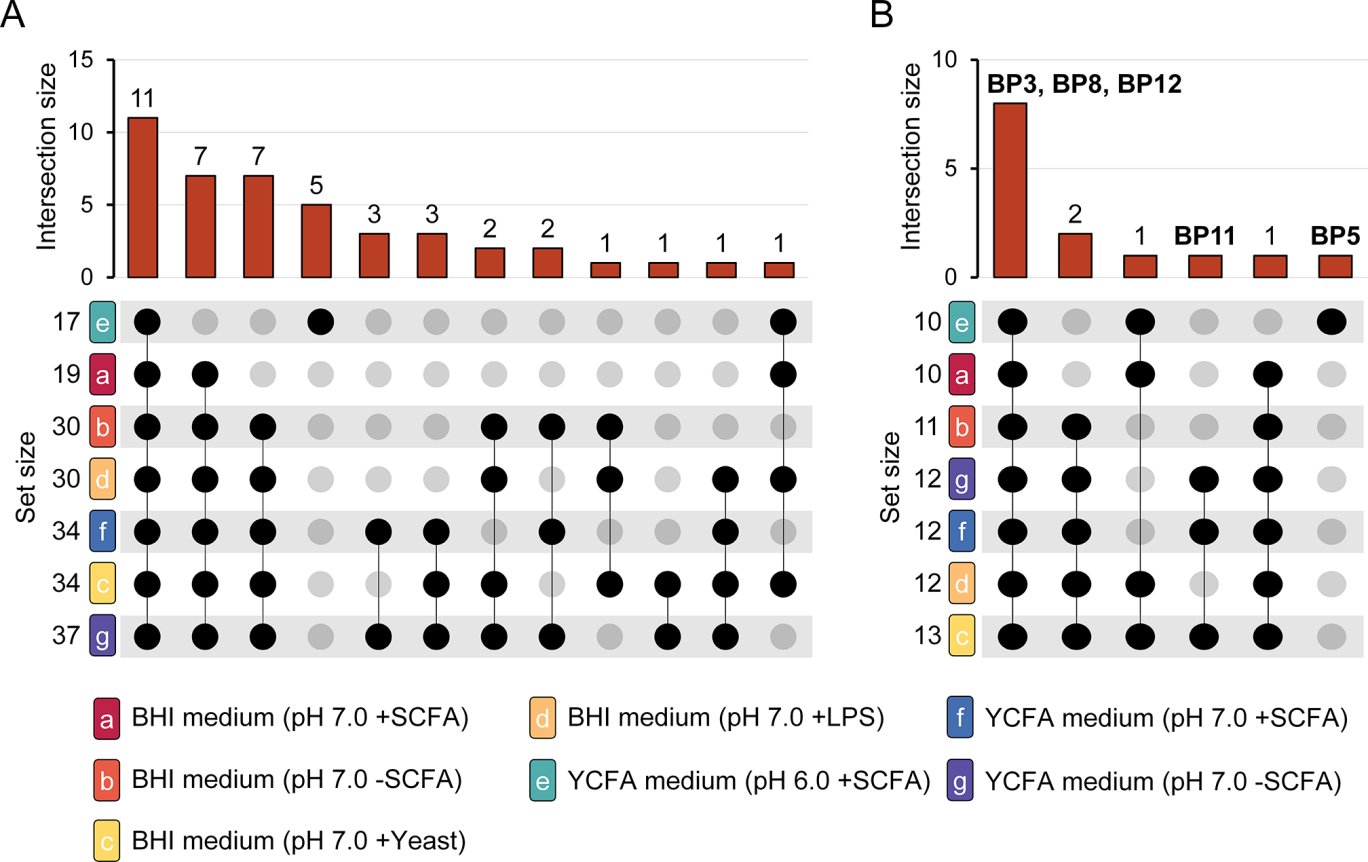
Identification of SEP in dependence of the composition of the culture media. (**A**) Upset plot analysis of novel SEP. (**B**) Upset plot analysis of previously described SEP, including those reported to be produced exclusively within the microbiome community, highlighted for reference. UpSet plots show overlap in bottom-up identifications across the seven different culture conditions, represented by different colors on the *y*-axis and sorted by increasing set size. Set size corresponds to the total number of SEP identified in each condition, and intersection size represents the number of shared SEP identified through different approaches.

Interestingly, 11 of the 44 SEP were detected in all 7 growth conditions (Fig. 2A), suggesting that these SEP play a universal role in *B. producta* and may be essential for the survival and growth of *B. producta* under different environmental conditions.

Seven SEP were identified in all culture conditions except for the acidic YCFA condition (pH 6.0 + SCFA). Interestingly, the opposite was observed for a specific group of five SEP, with their presence being observed only under acidic conditions. Furthermore, we found that several SEP could only be identified in the presence of cellular components such as yeast extract or lipopolysaccharide. These results suggest that extracellular factors, such as pH or the presence or absence of endotoxins (i.e., LPS), can influence the production of SEP. Nevertheless, some SEP were produced independently of these factors, including the production of three SEP in YCFA medium (pH 7.0), which were detectable with or without the presence of SCFAs. Note that the presence of certain SEP in other conditions cannot be ruled out, as they may be present at concentrations below the limits of detection or may be obscured by other factors.

Overall, our BUP analysis identified all of the previously described SEP (3), with the exception of BP15. Notably, BP15 was uniquely identified using an Asp-N protease approach (3), highlighting the importance of using multi-protease approaches for SEP discovery (31). Furthermore, five SEP (BP3, BP5, BP8, BP11, and BP12) were identified only during co-culture with other bacteria (SIHUMIx). In contrast, our data from single-bacteria cultivations suggest that the biosynthesis of these SEP is not dependent on interspecies interactions or communication within the microbiome. Furthermore, our results suggest that the production of several SEP may be influenced by specific bacterial growth and stress factors (Fig. 2B). For example, BP11 appeared to be linked to the presence of yeast extract in the medium, although it was not detected in YCFA medium at pH 6.0. In contrast, BP5 was only detected at pH 6.0, indicating a potential involvement in the acid stress response. Moreover, the SEP BP3, BP8, and BP12 previously identified only under coculture conditions were discovered to be not restricted to particular growth conditions.

#### SEP identified by top-down proteomics

**Fig 3:**
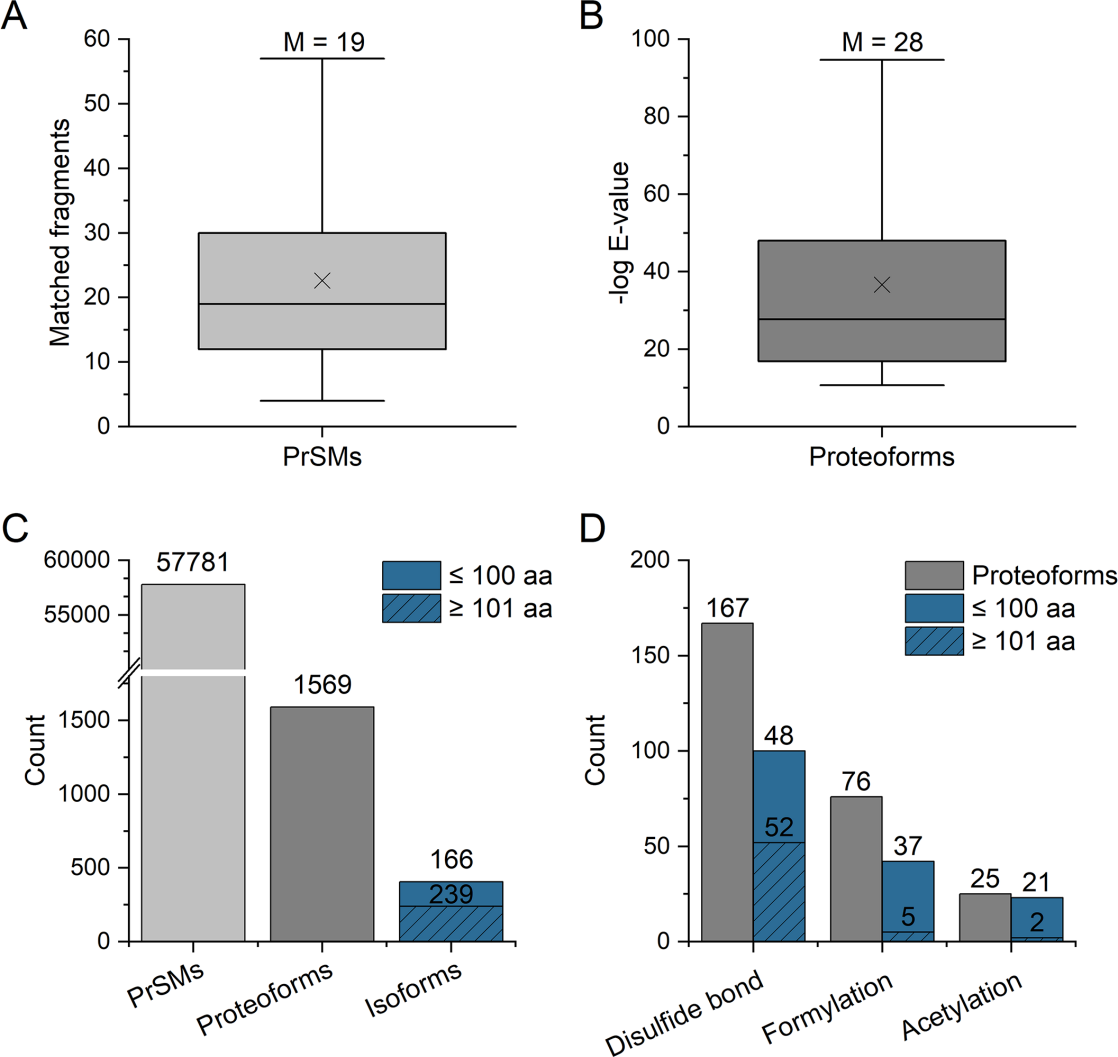
Proteomic properties of the top-down data set. (**A**) Box-and-whisker plots of number of matched fragment ions. (**B**) Box-and-whisker plots of −log *E*-values of proteoform identifications. (**C**) Bar plots of the number of identified PrSMs, proteoforms, and isoforms, between proteins with 100 or fewer or more amino acids. (**D**) Proteoforms and isoforms identified with modifications. Box-and-whisker plots capture lower quartile and upper quartile with median displayed as a horizontal line and mean depicted as a cross; whiskers represent minimum and maximum values that fall within 1.5 times the interquartile range. The value above the plot represents the median.

TDP analysis targeting small soluble proteins from *B producta* identified 166 small proteins (less than 101 aa) (Fig. 3C) of which 19 SEP (in 55 proteoforms) were confidently identified (Tables S3 and S4). Further, the data set provided evidence of over 239 proteins and 1,569 proteoforms deriving from the proteomes of the pH 7.0 (1,224 proteoforms) and the pH 6.0 YCFA samples (131 proteoforms), respectively. The proteoforms were identified with a median number of 19 fragment ions, and 25% were identified with 12 or fewer matched fragment ions (Fig. 3A). The median *E*-value (1*E*−28) provides strong evidence for a high confidence of the proteoform identifications (Fig. 3B).

Seventeen of the 19 SEP were detected either as full-length or as N-terminal methionine excision (NME) proteoforms, with an average residue cleavage of around 50%. While 17 of the identified SEP were also detected in the pH 7.0 and the pH 6.0 YCFA samples in the BUP data sets, BP24 could not be unambiguously identified in the BUP samples, as due to its high frequency of lysine or arginine residues, only a single tryptic peptide could be detected. The SEP BP46 was exclusively identified using TDP.

The high number of full-length SEP proteoforms identified through high-quality intact protein mass spectra data, in conjunction with their simultaneous identification via BUP, provides compelling evidence for their existence. Furthermore, the identification of BP3, BP8, and BP12 delivers further hints that their expression is not dependent on the presence of interspecies communication.

Numerous proteoforms with N-terminal formylation, acetylation, or disulfide bonds were identified (Fig. 3D). Notably, this included the identification of SEP proteoforms. For example, three distinct proteoforms of BP3, including NME, NME with N-terminal acetylation, and NME with N-formylation (Fig. S3B), could be identified. Furthermore, both full-length and truncated proteoforms with N-terminal formylation and/or free amino termini were identified for BP4, BP7, and BP10 (Fig. S3). These data suggest that SEP, like all proteins, can undergo post-translational protein processing and that the modifications may impact its biological function. For example, N-terminal acetylation of proteins is known to have a substantial impact on various physiological processes, encompassing metabolism, transcription, translation, and virulence (32 – 34), and has also been associated with the development of certain diseases (35).

**Fig 4:**
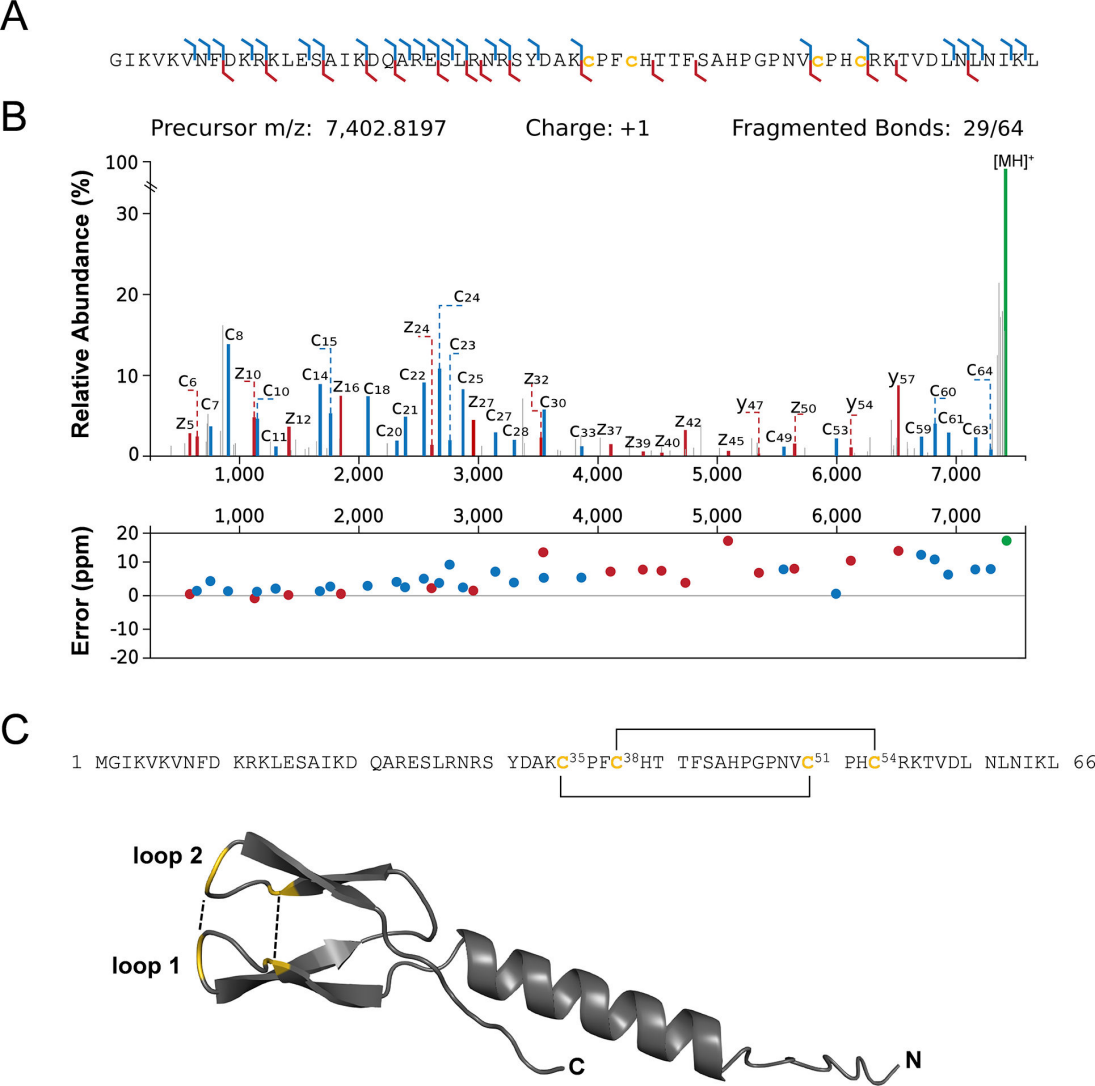
Identification and analysis of disulfide bonds in BP12. (**A**) The proteoform was identified with N-terminal methionine excision by 162 PrSMs. (**B**) MS^2^ spectra obtained after EThcD fragmentation provide evidence for the presence of two disulfide bonds. The proteoform sequence is annotated with the identified c- ions and y-, z-ions in blue and red, respectively. Neutral losses and other ions are not shown for clarity. The dot plot illustrates the mass error in ppm, confirming the correct identification for each observed ion. (**C**) Sequence and structural motif predictions of the mature BP12. The predicted disulfide bridges between Cys_35_-Cys_51_ and Cys_38_-Cys_54_ are indicated by lines connecting the orange-colored cysteine residues, with the positions of selected amino acids indicated by superscript numbers.

The top-down data provided strong evidence for disulfide bridges in three SEP (BP12, BP24, and BP46) (Table S4). Unfortunately, the exact assignment of these linkages to specific cysteine residues was not possible with our data up to now (Fig. 4A and B). Prediction of the secondary structure of BP12 using AlphaFold revealed the presence of both α-helix and β-sheet structures, as well as potential disulfide bonds between Cys_35_-Cys_51_ and Cys_38_-Cys_54_ (Fig.4C; Fig. S4), providing valuable information for further functional studies. The previously reported antibacterial properties for BP12 were based on AMP scores from AMP Scanner v.2 (36). However, AMPfun’s prediction applied in our study did not indicate any AMP activity for BP12. Differences in predictions can be explained by variations in the training sets used, their size, physiochemical properties, and the frequency of amino acids at each sequence position, which can affect the accuracy of the prediction model for classifying antibacterial peptides (37). As these predictions are inherently probabilistic in nature, it is crucial to validate the predictions experimentally to confirm the antimicrobial susceptibility and activities of BP12 in future studies.

**Fig 5:**
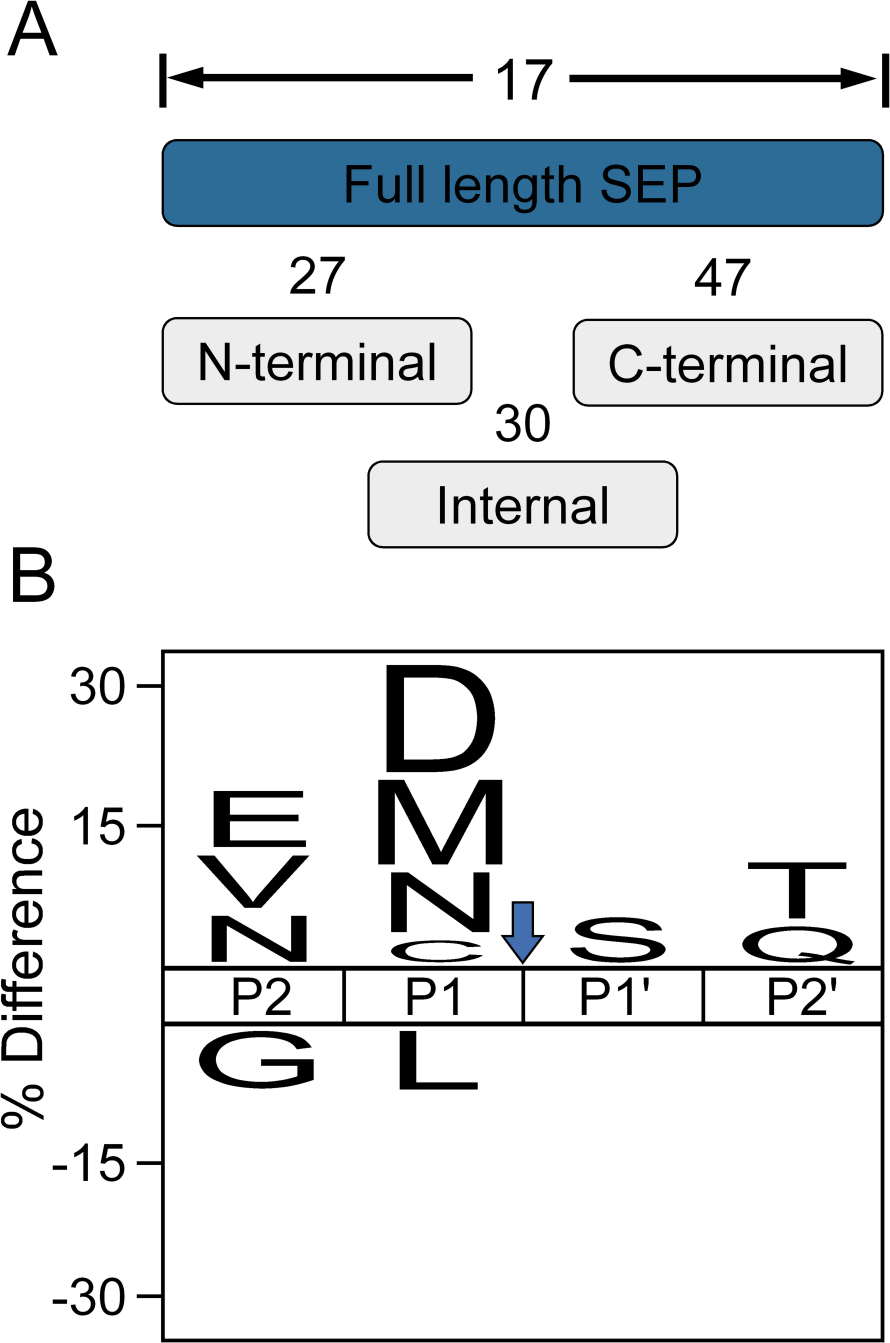
Top-down SEP proteoform diversity. (**A**) Distribution of identified SEP with a canonical N-terminus, including proteoforms that have been derived by N-terminal methionine excision (NME), proteoforms with a canonical N- or C-terminus, and internal proteoforms lacking either a canonical N- or C-terminus. (**B**) Differential proteoform truncation analyses, highlighting potential specificities for Asp/Asn and Met truncations. IceLogo plot illustrating the relative frequency of amino acids found at the P2-P2′ positions surrounding the truncation site (indicated by the blue arrow). Amino acids that are significantly enriched (top) or de-enriched (bottom) (*P* ≤ 0.05). Proteoforms derived by NME have been excluded prior to the analysis.

The unambiguous identification and characterization of both N- and C-terminal regions (canonical or neo-protein termini) of proteoforms are often not possible in BUP (19). In contrast, TDP directly provides information about both termini. Protein truncation has been shown to play an important role in many biological processes (38, 39). We identified several N- or C-terminally truncated SEP proteoforms (Fig. 5A) and evaluated potential truncation sites that may have resulted from endoprotease or exopeptidase activities or artificial truncation during sample preparation, displaying the positions surrounding the potential truncation site (P2-P2′) as an IceLogo plot (Fig. 5B). Although proteoforms resulting from NME were excluded from this analysis, we observed potential specificities for methionine in the P1 position, indicating the presence of methionine aminopeptidase (MAP) activity. The rate of methionine excision is influenced by the size of the side chain of the P1’ amino acid, with small amino acid residues such as alanine, glycine, proline, serine, and cysteine exhibiting a high efficiency of methionine removal due to their small radius of gyration (40). This pattern is consistent with our observations and adds further evidence for the presence of MAP activity in the sample (Table S1). However, since MAP activities depend on N-methionine peptides with a free amino group (41), the presence of N-terminally truncated proteoforms with N-terminal methionine could hint toward an potential alternative translation start site as observed in BP4 and BP7 (Fig. S3C and E). The coexistence of two proteoforms, one potentially derived from alternative translation in the cell, suggests a potential functional significance.

Proteoforms with an N-terminal methionine, but lacking the encoded N-terminal, part were also detected, for example, BP14 and BP20, which initiate with a second methionine at positions 24 and 8, respectively (Fig. S3J and K). We utilized the Phobius algorithm to investigate the potential presence of a signal peptide in the full-length sequences of these proteins (42). The algorithm predicted a high posterior probability of 0.87 for an N-terminal signal peptide in BP14 and a posterior probability of 0.96 for a C-terminal non-cytoplasmic region. The absence of detection of the N-terminal sequence supports the notion that a signal peptide could have been cleaved. However, further research may be required to confirm this, as the possibility of alternative initiation or cleavage by a signal peptidase remains uncertain.

### Physicochemical properties of the identified SEP

Several physicochemical properties such as the isoelectric point (pI) and the grand average of hydropathy (GRAVY) score of the identified SEP were analyzed and compared with the reference proteome of *B. producta*. The pI values showed a bimodal distribution, with a shift of the SEP toward more basic pI (Fig. S5A). The distribution of GRAVY scores indicated a wider range of hydrophilic SEP (Fig. S5B). Additionally, the analysis of amino acid distribution revealed lower prevalence of hydrophobic amino acids such as alanine, leucine, isoleucine, and a higher frequency of lysine or arginine residues in SEP (Fig. S6). The high frequency of lysine or arginine residues may explain the high number of identifications made during this study, as these residues can improve proton affinity and ionization efficiency in mass spectrometry analysis, leading to increased detection sensitivity for these types of proteins (43). Given the fact that 71% of the identified SEP were predicted to be non-cytoplasmic, with BP14, BP37, and BP45 predicted to have signal peptides, one can hypothesize that these proteins may play a role in mediating cell-cell and cell-host communication (3, 44, 45). To identify potential mediators of homeostasis among bacteria, we used AMPfun (46) and AMP scanner v.2 (36), which predicted several SEP as antimicrobial peptides, with BP40 predicted to be capable of targeting both Gram-positive and Gram-negative bacteria (Table S1), making it a promising candidate for further studies on its potential as an antimicrobial agent.

The identified proteins possess typical biochemical characteristics, which aligns with our applied workflow that targets mainly soluble proteins. However, the potential impact of short transmembrane proteins, which were predicted to make up 35% of all SEP in the human microbiome (13), should not be ignored, as these proteins can interfere with cellular processes such as transport, signaling pathways, cell division, respiration, sporulation, and membrane integrity (8, 47). Therefore, future studies may focus on these membrane-associated SEP using specific and MS-compatible membrane protein enrichment protocols (18, 48), which could facilitate the identification of these proteins and their potential functions (49).

#### Expanding the annotation of the RefSeq database: discovery of novel ORFs and proteoform variants

Using our BUP data, the capabilities of iPtgxDB were utilized to expand the knowledge of known coding sequences and their translational products by identifying novel translation products that had not been previously documented in the RefSeq database of *B. producta*. Employing the rigorous filtering protocol, we identified 14 putative ORFs, of which seven were subsequently validated through the two-step verification process. The confirmed ORFs showed evidence for alternative protein translation initiation sites (41), resulting in variation of the N-termini, in C-terminal extensions when compared with their corresponding RefSeq proteins (Table 1). The high numbers of PSMs observed provided robust evidence to support these predictions.

**TABLE 1:** Summary of iPtgxDB results, including the RefSeq anchor annotation, genomic start and stop positions, reading frame, start codon, protein-coding sequence (CDS) length, and predicted sequence, with the RefSeq anchor start methionine highlighted in bold^*a*^.

*In silico* prediction	RefSeq anchor protein (accession; ORF name)	Genomic location (reading frame; start codon)	#Peptides (PSMs)	Extension (CDS length)	Predicted sequence
Chem- Genome	30S ribosomal protein S1 (A0A4P6M2B6; E5259_11800)	2646213–2647325 (+3; TTG)	1 (10)	N-term +6 aa (370 aa)	MRRFQN**M**
	Bacteriophage protein (A0A7G5N3A9; E5259_08725)	1980586–1981020 (+1; GTG)	5 (263)	C-term +68 aa (144 aa)	MDQSLKGEYIMATTK KSQFKINNGTDWDTYHFETDSAQVKHKKSDGTETTVEDVLNSTLGGYMIQAGR
	N-acetylmuramoyl-L-alanine amidase (A0A7G5N3D3; E5259_10710)	2412738–2413589 (+3; CTG)	4 (137)	N-term +151 aa (283 aa)	MTLRRIVIAFLLKGGRRMNKWLKKGILLLAVGCLTFAVTGCSGEKVMDKVLEKTDPKPDNTEKNSDKEEENKTQEPEVEVAKPEFTANLSGSVTYKTGDKAEALKVEAKTSDKGVITYQWYQSQTNTNGGGTPVEGETKNTFTPPTGEAKT**M**
	Peptidase_S8 domain-containing protein (A0A7G5N366; E5259_05365)	1228334–1227327 (−2; ATG)	3 (95)	N-term +199 aa (335 aa)	MCKFQILIQLKGGTIMGCLVFILFCIVIFALMMIAYMYAVGIIIAVDVISGIALLASVGSYINAKKMKPSTMQCPNCSNPNVKLSTIQTGGQRKRIANCQSCGFEYDYITPDDITQGKSKSIGLIAIFAIILAVGLTFTFRLLGSSSSPDSKSDAVVASTEEPDSEKVKMDDFEYSIDGETISLERYNGDSKILEISPT**M**
Prodigal	Cell division protein FtsZ (A0A7G5N396; E5259_07825)	1768310–1769509 (2, TTG)	1 (1013)	N-term +4 aa (399 aa)	MLEI**M**
	ABC transporter ATP-binding protein (A0A7G5N386; E5259_06750)	1516067–1517599 (+2; GTG)	1 (32)	N-term +7 aa (510 aa)	MEQYVIE**M**
	Translation initiation factor IF-3 (A0A4P6LZI1; E5259_22155)	4793551–4792910 (−3, ATG)	1 (124)	N-term +49 aa (213 aa)	MRICVDSRLSSIHFFLVSFRMFYEVEGRRAGQEQSVLSILWRCTTISDL**M**

^*a*^ Out of 31 non-canonical peptides, seven were excluded by PepQuery as they could not be matched, had better matches to other peptides, or carried potential posttranslational modifications. The remaining 24 peptides passed verification and met the statistical threshold. Further BLASTp analysis excluded eight more peptides.

**Fig 6:**
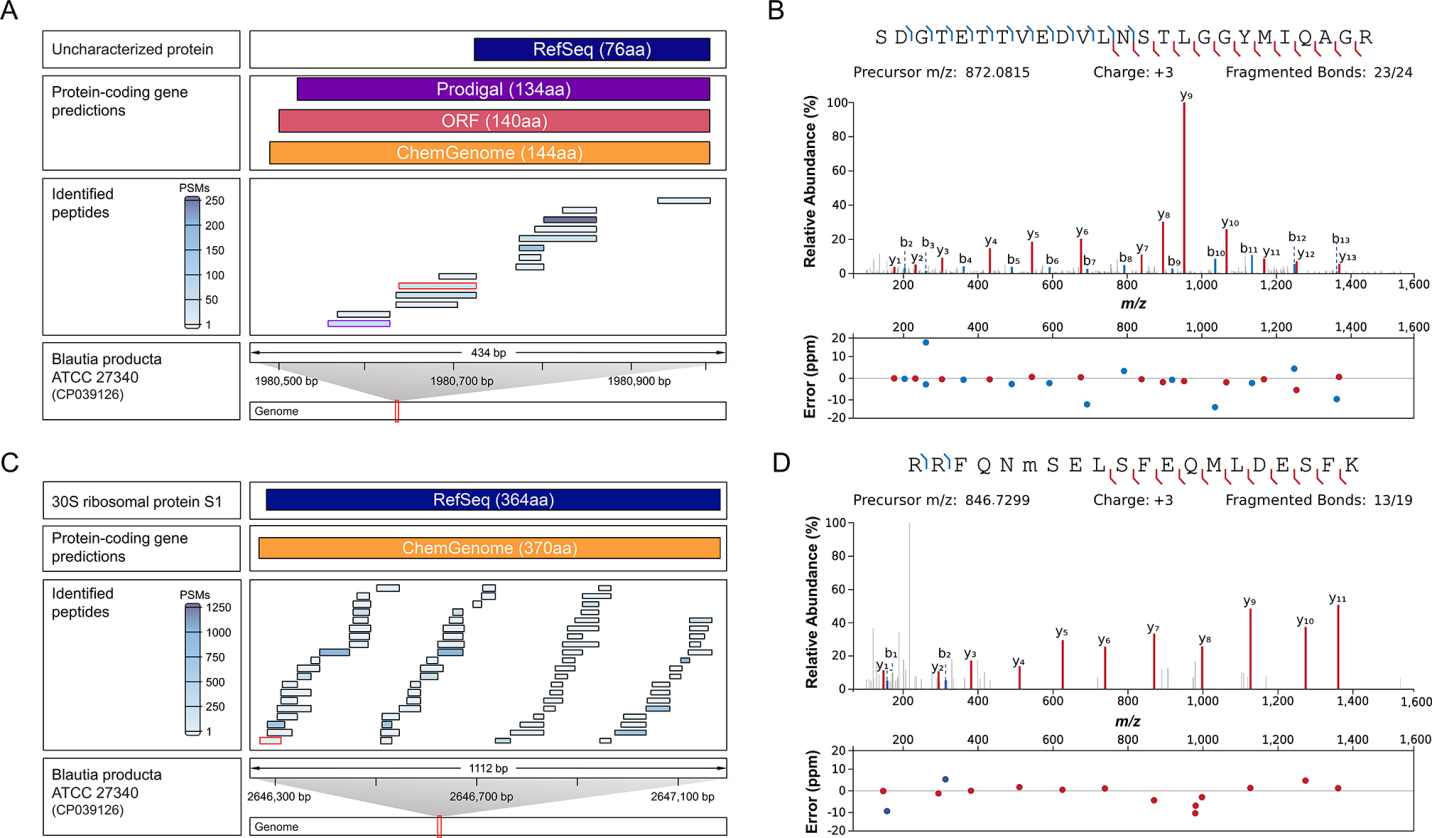
Identification of multiple peptides supports the proteogenomic predictions. Peptide-level evidence for (**A**) an N-terminal extension of the uncharacterized protein (located at 1,980,790–1,981,020) and (**C**) an alternative start site for 30S ribosomal protein S1 (located at 2,646,231–2,647,325). The respective genomic localizations of the corresponding RefSeq gene and proteogenomic annotations uncovered in the *B. producta* genome (CP039126) are depicted. Identified peptides are represented as blue horizontal lines and are shaded according to their estimated abundance, based on the number of PSMs. (**B**) PSM of the peptide SDGTETTVEDVLNSTLGGYMIQAGR (highlighted in red), which supports the longer N-terminal proteoform, was identified with 71 PSMs. (**D**) PSM of the peptide RRFQNMSELSFEQMLDESFK (highlighted in red), with Met6 being oxidized, is presented with 11 PSMs, providing evidence for the longer alternative start site. PSM of the highlighted peptide in purple can be found in Fig. S7. Peptide sequences are annotated with the identified b- and y-ions in blue and red, respectively. Neutral losses (−H_2_O, −NH_3_, and −CO_2_) and other ions than b- and y-ions are omitted for clarity. The dot plots illustrate the mass error in ppm, confirming the correct identification for each observed ion.

For instance, for the “uncharacterized protein, A0A7G5N3A9,” Prodigal, ORF, and ChemGenome predicted a N-terminal extension of 58 to 68 amino acids compared with that for the annotated RefSeq protein (Fig.6A). Multiple peptides spanning internal regions (Fig.6B; Fig. S7) of these newly predicted proteoforms could be identified. However, the high frequency of lysine residues in the N-terminal part of this protein prevented the identification of N-terminal peptides (Table 1), preventing to determine the true length of the transcript. The identification of an alternative start site for the 30S ribosomal protein S1 (A0A4P6M2B6) resulted in the prediction of a proteoform N-terminally prolonged by six amino acids compared with the RefSeq protein (Fig. 6C); its presence could be confirmed by the identification of one peptide (Fig. 6D).

### Conclusions

The inclusion of a wide range of growth conditions, and the combination of BUP and TDP approaches, allowed for the identification of 45 SEP, 31 of which are described for the first time. While BUP was, due its sensitivity, very efficient for inference-based identifications of the majority of SEP, extensive control measures were performed to improve the confidence of identifications. An important consideration in the identification of SEP comes from the inherently higher FDR associated with large databases (30, 50), which may have been a critical factor limiting the depth of previous analyses (3). These factors further justify the use of the stringent filtering regimes employed here (i.e. peptide-centric database validation and ion series inspection) which have recently also been recommended for the field (51).

The parallel use of TDP helped to further validate the proteins identified via BUP and additionally revealed a SEP not detected via the BUP workflow. Furthermore, the TDP analysis enabled the identification of a number of proteoforms, which includes several post-translational modifications and truncation events. The latter may derive from proteolytic processing or be the consequence of the use of alternative initiation sites in their biosynthesis. By further exploring the possibilities of the iPtgxDB, we were able to identify seven new ORFs corresponding to RefSeq proteins, highlighting the database’s potential to identify novel translation products and proteoform variants of known protein-coding sequences. These results emphasize the importance of incorporating a proteogenomic approach for the comprehensive annotation of protein-coding genes to expand our understanding of proteome complexity.

Most strikingly, our data show that SEP previously described to be produced exclusively in co-culture systems can also be produced in monoculture conditions, indicating that their production is not entirely dependent on interspecies interactions. We found that different growth conditions and extracellular factors, such as pH and the presence of endotoxins (LPS), can stimulate/regulate the production of certain SEP, suggesting that SEP may also play a role in host-microbiome interactions, e.g., in chronic inflammatory bowel diseases (IBD). To address the functional properties of SEP and elucidate their clinical relevance, further investigations, e.g., targeting bacteria-bacteria interactions, as well as improvements in the sensitive of techniques for detection of SEP, are needed. Additionally, while this analysis focused on the identification of SEP across a range of growth and stress conditions, to further investigate biological function and mechanistic roles, future work incorporating quantification strategies will be of immense value. If combined with further refined databases, generated though the interrogation of gene-level information (e.g. RiboSeq and ribosome profiling) (50), it will be possible not only to delve deeper into the proteome of these elusive small proteins but to also elucidate biological functions of the SEP.

## MATERIALS AND METHODS

### Bacterial strains and growth conditions

YCFA medium was prepared according to the respective recipe from the German Collection of Microorganisms and Cell Cultures GmbH (DSMZ, Leibniz Institute, DSMZ Media 1611) and adjusted to either pH 6.0 or pH 7.0. Modifications were made for experimental comparison omitting the SCFAs from the media preparation. BHI medium (Carl Roth) was prepared accordingly and adjusted to pH 7.0. The BHI media were supplemented with either 2.5 mg/mL yeast extract, 100 ng/mL LPS (*E. coli* O128:B12, Sigma Aldrich), or the amount of SCFA corresponding to the original YCFA media. *Blautia producta* strain DSM 2950 was purchased from DSMZ, and constant maintenance culture was performed on Chocolate agar PolyViteX (Biomérieux) at 37°C. All cultures were incubated under anaerobic conditions in an anaerobic chamber (H35, Don Whitley Scientific Limited, UK) with 85% (vol/vol) N_2_, 10% (vol/vol) CO_2_, and 5% (vol/vol) H_2_. An overnight culture from a single colony of *B. producta* was prepared in 5-mL liquid YCFA medium in a Hungate Tube without shaking. Growth curves were conducted as follows: a volume of 200 µL per medium type was prepared in five replicates in a 96-well Microtiter plate (Greiner 96 flat bottom) with medium only as control. Each well was inoculated with 0.1% (vol/vol) of the overnight culture. Subsequently, the plate was sealed with petroleum jelly and parafilm to ensure anaerobic conditions outside the anaerobic chamber. Bacterial growth was measured every 20 min (600-nm) for 71 hr in a multiplate reader (BioTek, Epoch 2), with continuous shaking at a frequency of 237 cpm at 37°C. Data were collected and exported using the Gen5 program (BioTek). For proteome analysis, 25-mL cultures of the respective media were inoculated with 0.1% (vol/vol) of the overnight culture and grown under anaerobic condition as outlined above. After bacteria reached the exponential phase, they were sedimented by centrifugation (7,000 × *g*, 10 min, 4°C) and resuspended in lysis buffer (6 M Guanidine Hydrochloride (GndHCl), 100 mM HEPES, 20 mM NaCl, and 1× complete protease inhibitor [Roche], pH 7.5).

### Protein extraction and preparation

*Protein extraction* – Bacterial cells were extracted by disruption through 10 freeze-thaw cycles (−80°C ethanol bath for 30 s, followed by sonication bath thawing for 30 s). The resulting cell debris was removed by centrifugation (21,000 × *g*, 20 min, 4°C), and the remaining supernatant was collected and combined with the previous sample after being washed twice with the lysis buffer. Protein concentrations of the extracted proteins were determined by BCA assay (Thermo Fisher Scientific), and proteins were aliquoted for full-proteome analysis (50 µg) or for the depletion protocol (400 µg).

*Bottom-up full-proteome analysis –* Extracted proteins (50 µg) were reduced with 10 mM dithiothreitol (DTT) (56°C, 800 rpm, 1 hr) prior to alkylation with 55 mM iodoacetamide (RT, 800 rpm, 30 min). Afterward, samples were precipitated by ethanol, suspended in 200 µL digestion buffer (0.1 M TEAB, pH 8.5), and digested using trypsin (Promega) with a protease-to-protein ratio of 1:40 (wt/wt). After incubation for 20 hr at 37°C, samples were acidified to pH 2–3 with 10% trifluoracetic acid (TFA), desalted using solid-phase extraction, and freeze dried. Peptide samples were suspended in loading solution (3% ACN and 0.1% aqueous TFA) for subsequent measurements.

*Depletion of high-molecular weight proteins –* To deplete the majority of high-molecular weight proteins and increase the identification of low-molecular weight (LMW) proteome, a total of 400 µg of extracted proteins were precipitated by ethanol and suspended in 80 µL of either 210 mM NaCl (acidic depletion mixture, final concentration of 50 mM NaCl and 0.1% TFA) or 420 mM TEAB (basic depletion mixture, final concentration of 100 mM TEAB) (22). The samples were vortexed to ensure solubilization and a 3.2× volume of ACN, with the addition of 0.1% TFA for the acidic depletion. After incubation for 1 hr at 20°C at 1,300 rpm, samples were centrifuged at 21,000 × *g* for 20 min at 20°C. The supernatant was then transferred to a new tube and dried using vacuum centrifugation.

*LMWP Bottom-up preparation –* Proteins were suspended in 200 µL of digestion buffer (0.1 M TEAB, pH 8.5), reduced, alkylated, trypsinized, desalted, and suspended in loading solution prior to LC-MS/MS analysis.

*LMWP top-down preparation –* Proteins were suspended in loading solution, mixed thoroughly, sonicated, and centrifuged (21,100 × *g* for 5 min). The supernatant was then transferred to an LC vial for further analysis.

### LC-MS/MS analysis

*Bottom-up –* Liquid chromatography-tandem mass spectrometry (LC-MS/MS) analysis was performed on a Q Exactive plus mass spectrometer coupled to an Dionex U3000 UHPLC (both Thermo Fisher Scientific). Peptides were concentrated onto a reverse-phase trap column (Acclaim PepMap100, 2 µm, 100 Å, 75 µm × 20 mm) with 30 µL/min for 5 min using 2% ACN and 0.05% TFA before being separated with a linear 120-min gradient from 95% eluent A (0.1% formic acid [FA]) to 50% eluent B (80% ACN, 0.1% FA) at a flow rate of 300 nL/min, on a C18 reversed-phase analytical column (Acclaim PepMap100, C18, 2 µm, 100 Å, 75 µm × 500 mm) (both Thermo Fisher Scientific). The mass spectrometer was operated in a positive ion mode. MS data were acquired using a data-dependent top10 method dynamically choosing the most abundant precursor ions from the survey scan (300 to 1800 m/z, resolution: 70,000, automatic gain control (ACG) target: 3e6, maximal injection time (IT): 100 ms) with a 2.0 m/z isolation window for high-energy collisional dissociation (HCD) fragmentation. Ions with unassigned and charge states +1 and >+8, respectively, were excluded. HCD spectra (resolution: 17,500, AGC target: 1e5, maximal IT: 100 ms, dynamic exclusion: 40 s) utilized a normalized collision energy (NCE) of 27. Three technical replicates were used to measure the full proteome samples, whereas two technical replicates were employed for measuring the LMWM samples. For each *in vitro* culture, two biological replicates underwent full-proteome and depletion analysis. However, the YCFA pH 6.0 condition was processed only with full-proteome analysis and was therefore prepared as five biological replicates.

*Top-down –* LC-MS/MS analysis was performed on a Orbitrap Fusion Lumos Tribrid mass spectrometer coupled to an Dionex U3000 UHPLC (both Thermo Fisher Scientific). Proteins were concentrated onto a reverse-phase trap column (PepMap300, C4, 5 µm, 300 Å) with 30 µL/min for 5 min using 2% ACN and 0.05% TFA before being separated with a linear 60-min gradient from 85% eluent A (0.1% aqueous FA) to 60% eluent B (80% ACN, 0.1% FA) at a flow rate of 300 nL/min, on an C4 reversed-phase analytical column (Accucore, 50 cm × 75 µm, 2.6 µm, 150 Å) (both Thermo Fisher Scientific). The mass spectrometer was operated in a positive ion mode equipped with the field asymmetric ion mobility spectrometry (FAIMS) pro interface utilizing a multi-compensation voltage (CV) method (52). Full-scan MS were acquired (400 to 1,800 m/z, resolution: 120,000, 30% RF lens, 200% ACG target, maximal injection time: 246 ms) using 15 V source-induced dissociation. Within a cycle time of 3 s, MS^2^ spectra were acquired with an isolation window of 5 m/z, 50,000 resolution, 400% AGC target, 250 ms injection time, and for fragmentation, collision-induced dissociation (CID) with an NCE of 30% was utilized. Only precursors with a charge state between 4 and 50 or undetermined charge states were selected with enabled dynamic exclusion (*n* = 2, 60 s). Four different CVs (–60, –50, –40, and –20 V) with different MS^1^ and MS^2^ settings were applied. Settings for CVs −60 and −50 V are as follows: resolution 60 k/50 k (MS^1^/MS^2^), maximum injection time 118/125 ms, microscans 2/2, AGC target 200%/400%. Settings for CVs –40 and –20 V are as follows: resolution 120 k/60 k, maximum injection time: 246/250 ms, 4/4 microscans. Acquisition was performed in a “peptide mode,” according to the recommendations of vendors for the mass range below ca. 20–30 kDa. Each sample was analyzed two times by LC–MS/MS. To more accurately assess the presence and connectivity of disulfide bonds, a reduction was performed on two samples using DTT (56°C, 800 rpm, 1 hr). An inclusion list for the targeted analysis of BP12 was generated for the orthogonal use of electron-transfer/higher-energy collision dissociation (EThcD) and CID fragmentation to increase residue cleavage.

### Data processing

*Bottom-up* – Processing of MS/MS raw spectrum data files was performed in Proteome Discoverer (PD) (version 2.5, Thermo Fisher Scientific) using the integrated proteogenomics search database (iPtgxDB) (16) for *Blautia producta* ATCC 27340 (accessed 2021.11.28) and 47 sequences of commonly observed laboratory contaminants. Raw files of the LMW proteome were combined into a single database search. Enzyme specificity was set to semi- and full-tryptic specificity allowing up to four missed cleavages (53). Carbamidomethylation of cysteine was set as a fixed modification and oxidation of methionine as a variable modification. The “match between runs” function was disabled, meaning that each peptide being analyzed must be identified with a unique PSM. The percolator algorithm was used for posterior error calculation (54) combining the results of the database searches restricted by a *q*-value to FDR ≤ 0.01, and protein groups were assigned using strict parsimony rules.

An additional filtering was implemented including the following: (i) the manual inspection of putative non-annotated peptide matches (27), (ii) the requirement that an SEP identification must have a sequence tag of at least five consecutive b- or y-ions in its MS^2^ spectrum (28), and (iii) the requirement that Prodigal and ChemGenome predictions must have at least three PSMs and *in silico* ORF predictions must have at least four PSMs (23).

*Top-down –* Database search was conducted using ProSightPD 4.0 nodes (Proteinaceous Inc., Evanston, USA) within Proteome Discoverer (version 2.5.0.400, Thermo Fisher Scientific), utilizing iPtgxDB. The high/high cRAWler node was employed for the automated generation of experiments in combination with Xtract for deconvolution. Acetylation and formylation at the N-terminus were treated as variable modifications. The Annotated Proteoforms Search, with a maximum of three proteoform spectrum matches (PrSMs) per precursor, a minimum of three matched fragments, and no delta M mode, was utilized to identify full-length proteoforms. Two Subsequence Search Nodes, each with a maximum of one PrSM per precursor, a minimum of six matched fragments, and a precursor and fragment mass tolerance of 10 ppm, were used to detect truncated proteoforms. One of these search nodes was specifically designed to search for fixed dehydro-modifications on cysteine in order to identify potential disulfide bridges. The TDP results were processed in a single multiconsensus step, filtering PrSMs and proteoforms with a false discovery rate (FDR) cut-off of 1%. To ensure accurate identification and validation of SEP, the related proteoforms must have a minimum C-Score of 40 (24), an *E*-value smaller than 1 × 10^–5^, and at least 10 matching fragment ions with a mass error less than 10 ppm (25, 26). Furthermore, SEP identifications had to meet the requirement of at least three PrSMs for Prodigal and ChemGenome predictions and at least four PrSMs for Silico ORF predictions (23).

### Bioinformatics analysis of BUP data

*Bottom-up validation* – To further validate SEP identifications, the standalone version of Pepquery (v. 2.0.2) (29) was employed. This peptide-centric search engine was used to confirm the quality of the PSMs and to determine if the matched sequences were unique or could be attributed to the *B. producta* proteome. The retrieved MS/MS spectra were converted to MGF format using msconvert from proteowizard (55). The final PepQuery command line appeared as follows: java -jar pepquery-2.0.2.jar -db [fasta file] -ms [mgf files] -i [peptid list] -aa -hc TRUE -c 4 -maxVar 4 -itol 0.02 -o [output directory], which allowed for unrestricted modification searching and substitution of amino acids. The results were then filtered to eliminate any peptides that matched the reference proteomes, leaving only those peptides that aligned with potential SEP. The validated peptides were then analyzed using the Proteomics Data Viewer (56) and further evaluated using NCBI Protein Blast against the *B. producta* proteome. The peptides and their associated PSMs with a *P* value of ≤0.01 that passed through this rigorous filtering process were used to create a high-confidence list, serving as the basis for identifying SEP.

*NCBI Protein Blast* – BLASTp search was performed using the NCBI protein RefSeq database (December 30, 2022). The search criteria for non-canonical peptides included an *E*-value threshold of 1 × 10^–5^, a minimum sequence identity of 90%, and a query coverage of 100%. The reference database used for comparison was the *B. producta* proteome (taxid:33035). To determine sequence conservation, the RefSeq database was searched for bacterial species (taxid:2) using the same *E*-value threshold, a minimum sequence identity of 50%, and query coverage of 50%. The closest relative hit to the query strain was reported when multiple homologs were identified.

*In silico analysis* – Further analysis was performed using a range of computational tools, including ProtParam for the calculation of physicochemical properties such as the isoelectric point (pI), grand average of hydropathy score (GRAVY) (57), and Phobius for the prediction of protein localization (42) (posterior probability ≥ 0.5). The potential antimicrobial peptide (AMP) activity and their functional targets were assessed using AMPfun and AMP scanner v.2 (a probability score of >0.5 indicates potential AMP activity, while a score < 0.5 indicates non-AMP activity) (36, 46). Disulfide bridges were predicted using SCRATCH (58). Functional domains and motifs were predicted using NCBI’s Conserved Domains search (v. 3.20) (59) and eggnog-mapper (v 2.1.9) (60). An additional functional characterization was performed using STRING (v 11.5) (61). Protein crystal structure predictions were generated using AlphaFold Colab v2.3.0 (62) and visualized using PyMOL (63). For *in silico* digestion, the online tool MS-Digest was employed to predict the peptides produced from proteolytic digestion. The UniProt reference proteome of *Saccharomyces cerevisiae* ATCC 204508 (accessed 2022.12.22, 6,060 entries), *Sus scrofa* (accessed 2023.03.25, 47,179 entries), *Bos taurus* (accessed 2023.03.25, 37,508 entries), and the iPtgxDB were used as inputs for the analysis. Trypsin was chosen as the proteolytic enzyme, with a maximum of two missed cleavages allowed and a minimum peptide length of seven amino acids.

The sequence coverage of BUP and TDP data was calculated using the protti package (64) in R (v.4.2.2). For cleavage site analysis, proteoforms with initiator N-terminal methionine excision, identical proteoform sequences with multiple modifications, and proteoforms with canonical C-termini were removed to eliminate false-positive cleavage sites. The normalized cleavage specificity was then calculated, taking into account the natural abundance of the corresponding amino acids in the *B. producta* proteome. Cleavage site specificity was visualized using IceLogo motif representations (65). UpSet plots were generated using the upsetplot and matplotlib package (66, 67) in Python (v. 3.11.1).

## Data Availability

The mass spectrometry proteomics data have been deposited to the ProteomeXchange Consortium (67) via the PRIDE partner repository with the data set identifier PXD041979.

## SUPPLEMENTAL MATERIAL

Supplemental file 1 spectrum.02528-23-s0001.pdf Fig. S1 to S7.

Supplemental file 2 spectrum.02528-23-s0002.xlsx Tables S1 to S4.

ASM does not own the copyrights to Supplemental Material that may be linked to, or accessed through, an article. The authors have granted ASM a non-exclusive, world-wide license to publish the Supplemental Material files. Please contact the corresponding author directly for reuse.
