# Supplementary material for: Identification of proteoforms of short open reading frame-encoded peptides in Blautia producta under different cultivation conditions
Source: Microbiol Spectr. 2023 Oct 2;11(6):e02528-23. doi: 10.1128/spectrum.02528-23 (PMC10715070; doi:10.1128/spectrum.02528-23)
Supplement: Supplemental file 1 — Fig. S1 to S7. [file spectrum.02528-23-s0001.pdf]

Supplemental Information

Identification of proteoforms of short open reading frame-encoded peptides in *Blautia producta* under different cultivation conditions

Jerome Genth[a#], Kathrin Schäfer[b#], Liam Cassidy[a], Simon Graspeuntner[b,c], Jan Rupp[b,c#], and Andreas Tholey[a*#]

[a]Systematic Proteome Research & Bioanalytics, Institute for Experimental Medicine, Christian-Albrechts-Universität zu Kiel, 24105 Kiel, Germany

[b]Department of Infectious Diseases and Microbiology, University of Lübeck, UKSH/ Campus Lübeck, Germany

[c]German Center for Infection Research (DZIF), Partner Site Hamburg-Lübeck-Borstel-Riems, Lübeck, Germany

<u>Contents</u>

Supplemental Figure 1: Growth of *B. producta* under different culture conditions.

Supplemental Figure 2: Analysis of potential peptide contaminations deriving from the use of protein-containing media.

Supplemental Figure 3: Examples of a SEP proteoforms identified by top-down proteomics.

Supplemental Figure 4: Structure prediction of the mature BP12 protein using Alphafold.

Supplemental Figure 5: Biochemical properties of the identified SEP

Supplemental Figure 6: Amino acid composition analysis.

Supplemental Figure 7: Peptide evidence supports the existence for an C-terminal extension of the referenced sequence of the uncharacterized protein (A0A7G5N3A9, E5259_08725).

Supplemental Table 1 – Supplemental Table 4: see separate Excel-file.

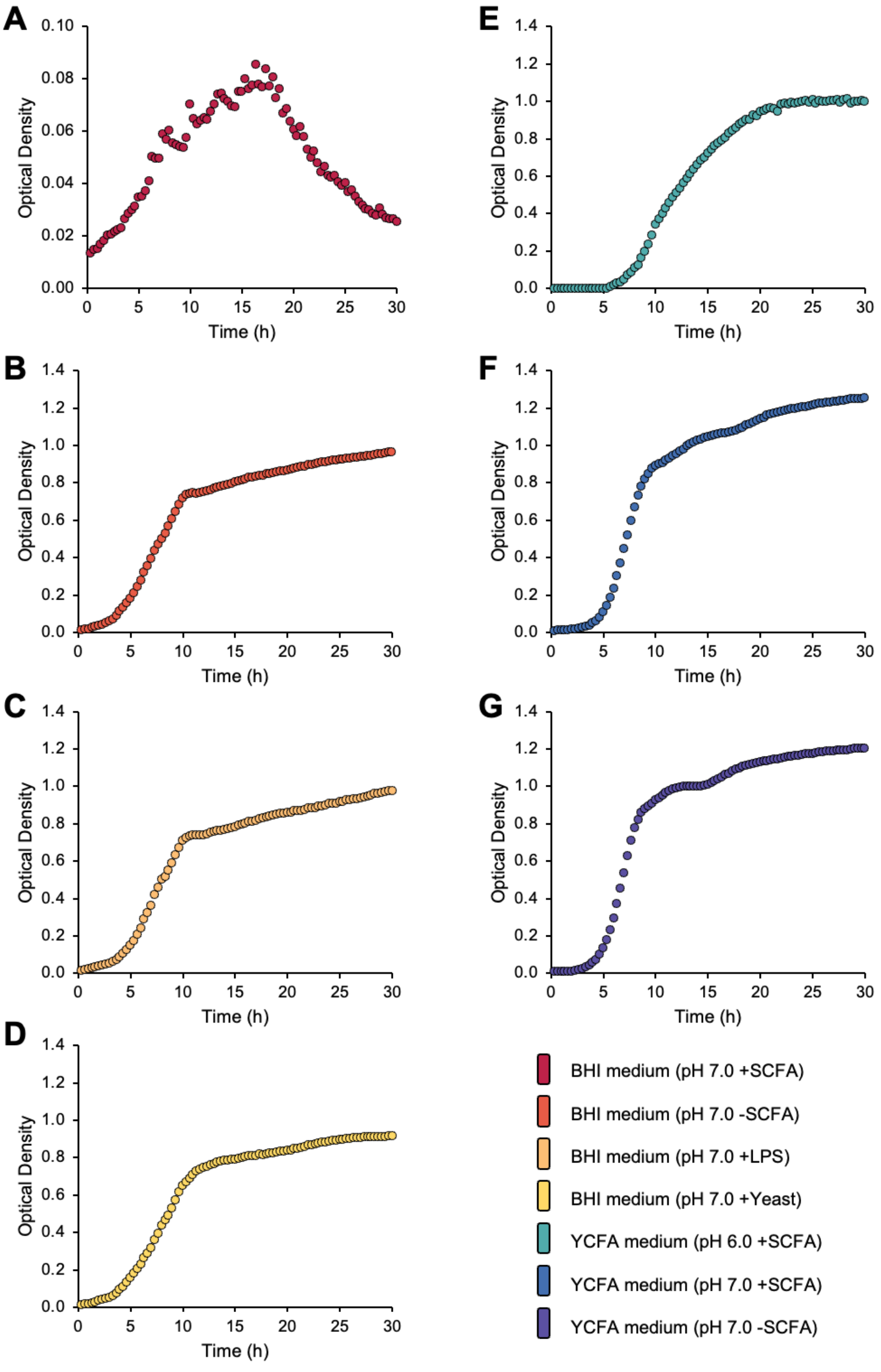

Supplement Figure 1. Growth of *B. producta* under different culture conditions. Growth with **(A)** BHI Medium (pH 7.0), **(B)** BHI medium (pH 6.0), **(C)** BHI medium (pH 7.0) substituted with 100 ng/ml LPS, **(D)** BHI medium (pH 7.0) substituted with 2.5 mg/ml yeast extract, **(E)** YCFA medium (pH 6.0) substituted with SCFA, **(F)** YCFA medium (pH 7.0) substituted with SCFA and **(G)** YCFA medium (pH 7.0) without SCFA.

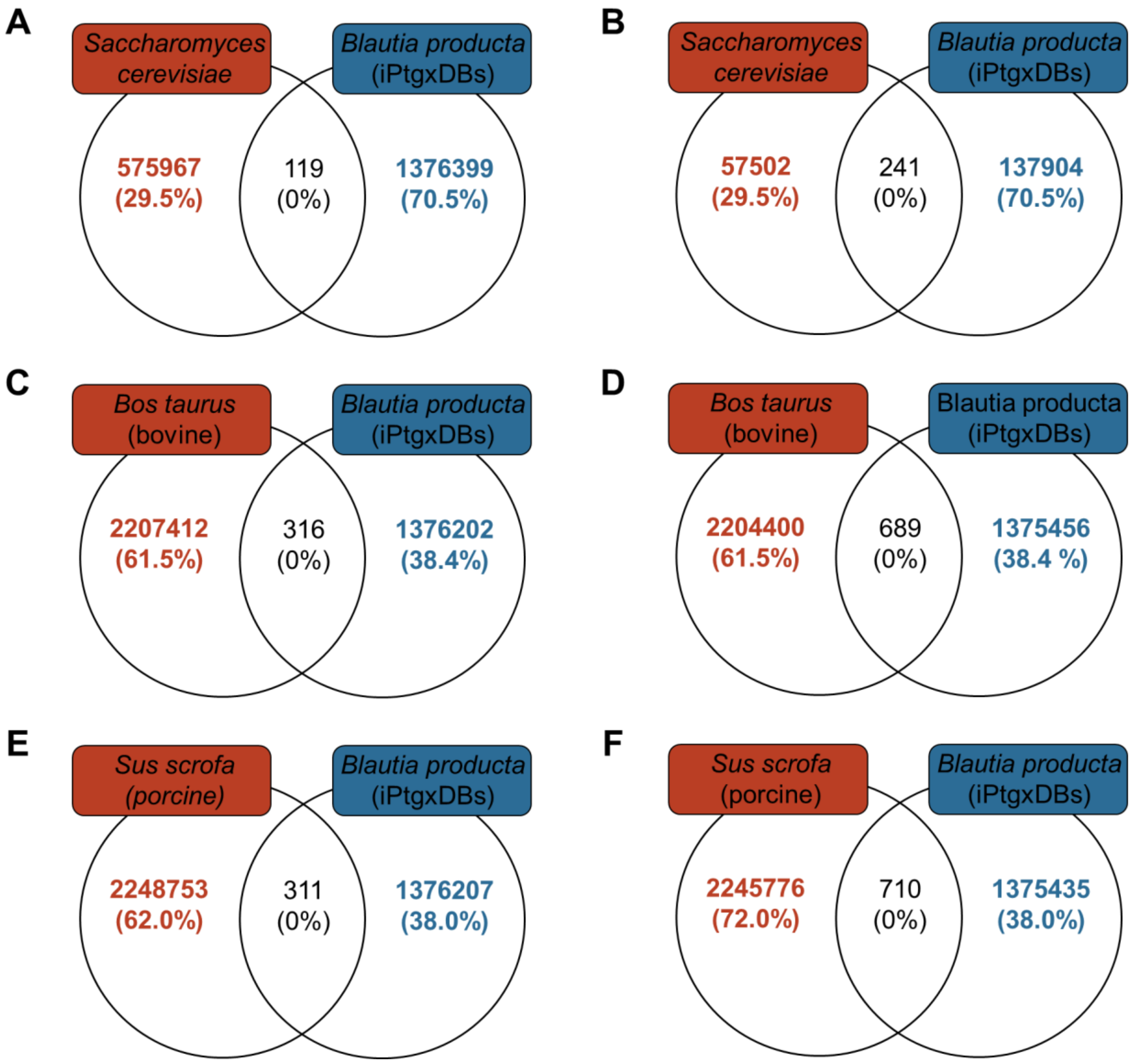

Supplemental Figure 2. Analysis of potential peptide contaminations deriving from the use of protein-containing media (yeast extract or Brain Heart Infusion). Overlap of identified peptides derived from *in-silico* tryptic digestion compared to the iPtgxDB of (*Saccharomyces cerevisiae, Sus scrofa and Bos Taurus*). Venn diagrams in **(A, C, E)** are based on 20-amino acids, while for **(B, D, F)** leucine and isoleucine were treated as a single amino acid.

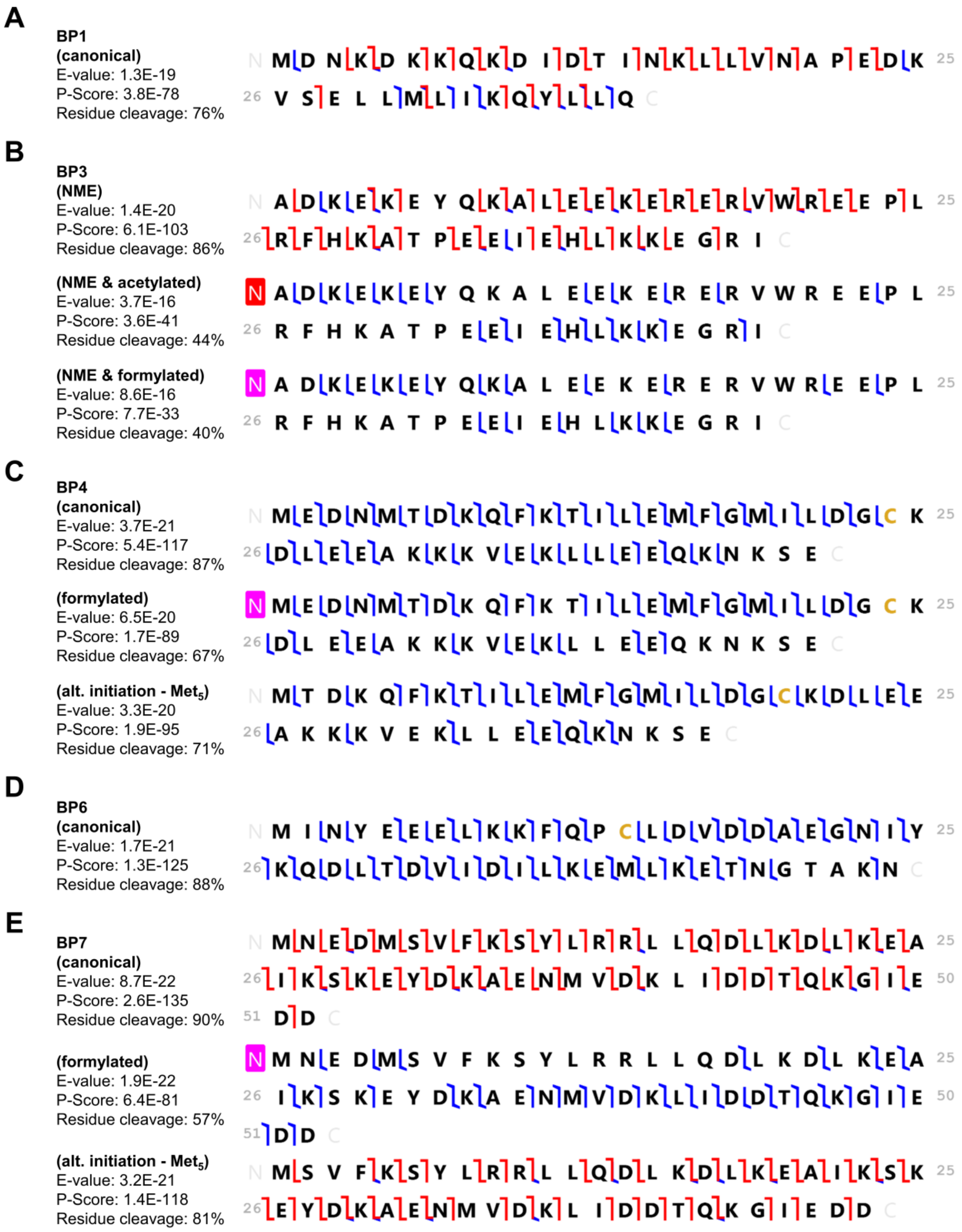
A
BP1
(canonical)
E-value: 1.3E-19
P-Score: 3.8E-78
Residue cleavage: 76%
N M D N K D K K Q K D I D T I N K L L V N A P E D K 25
26 V S E L L M L I K Q Y L L Q C
B
BP3
(NME)
E-value: 1.4E-20
P-Score: 6.1E-103
Residue cleavage: 86%
N A D K E K E Y Q K A L E E K E R E R V W R E E P L 25
26 R F H K A T P E E I E H L K K E G R I C
(NME & acetylated)
E-value: 3.7E-16
P-Score: 3.6E-41
Residue cleavage: 44%
N A D K E K E Y Q K A L E E K E R E R V W R E E P L 25
26 R F H K A T P E E I E H L K K E G R I C
(NME & formylated)
E-value: 8.6E-16
P-Score: 7.7E-33
Residue cleavage: 40%
N A D K E K E Y Q K A L E E K E R E R V W R E E P L 25
26 R F H K A T P E E I E H L K K E G R I C
C
BP4
(canonical)
E-value: 3.7E-21
P-Score: 5.4E-117
Residue cleavage: 87%
N M E D N M T D K Q F K T I L E M F G M I L D G C K 25
26 D L E E A K K K V E K L L E E Q K N K S E C
(formylated)
E-value: 6.5E-20
P-Score: 1.7E-89
Residue cleavage: 67%
N M E D N M T D K Q F K T I L E M F G M I L D G C K 25
26 D L E E A K K K V E K L L E E Q K N K S E C
(alt. initiation - Met5)
E-value: 3.3E-20
P-Score: 1.9E-95
Residue cleavage: 71%
N M T D K Q F K T I L E M F G M I L D G C K D L E E 25
26 A K K K V E K L L E E Q K N K S E C
D
BP6
(canonical)
E-value: 1.7E-21
P-Score: 1.3E-125
Residue cleavage: 88%
N M I N Y E E E L K K F Q P C L D V D D A E G N I Y 25
26 K Q D L T D V I D I L K E M L K E T N G T A K N C
E
BP7
(canonical)
E-value: 8.7E-22
P-Score: 2.6E-135
Residue cleavage: 90%
N M N E D M S V F K S Y L R R L L Q D L K D L K E A 25
26 I K S K E Y D K A E N M V D K L I D D T Q K G I E 50
51 D D C
(formylated)
E-value: 1.9E-22
P-Score: 6.4E-81
Residue cleavage: 57%
N M N E D M S V F K S Y L R R L L Q D L K D L K E A 25
26 I K S K E Y D K A E N M V D K L I D D T Q K G I E 50
51 D D C
(alt. initiation - Met5)
E-value: 3.2E-21
P-Score: 1.4E-118
Residue cleavage: 81%
N M S V F K S Y L R R L L Q D L K D L K E A I K S K 25
26 E Y D K A E N M V D K L I D D T Q K G I E D D C

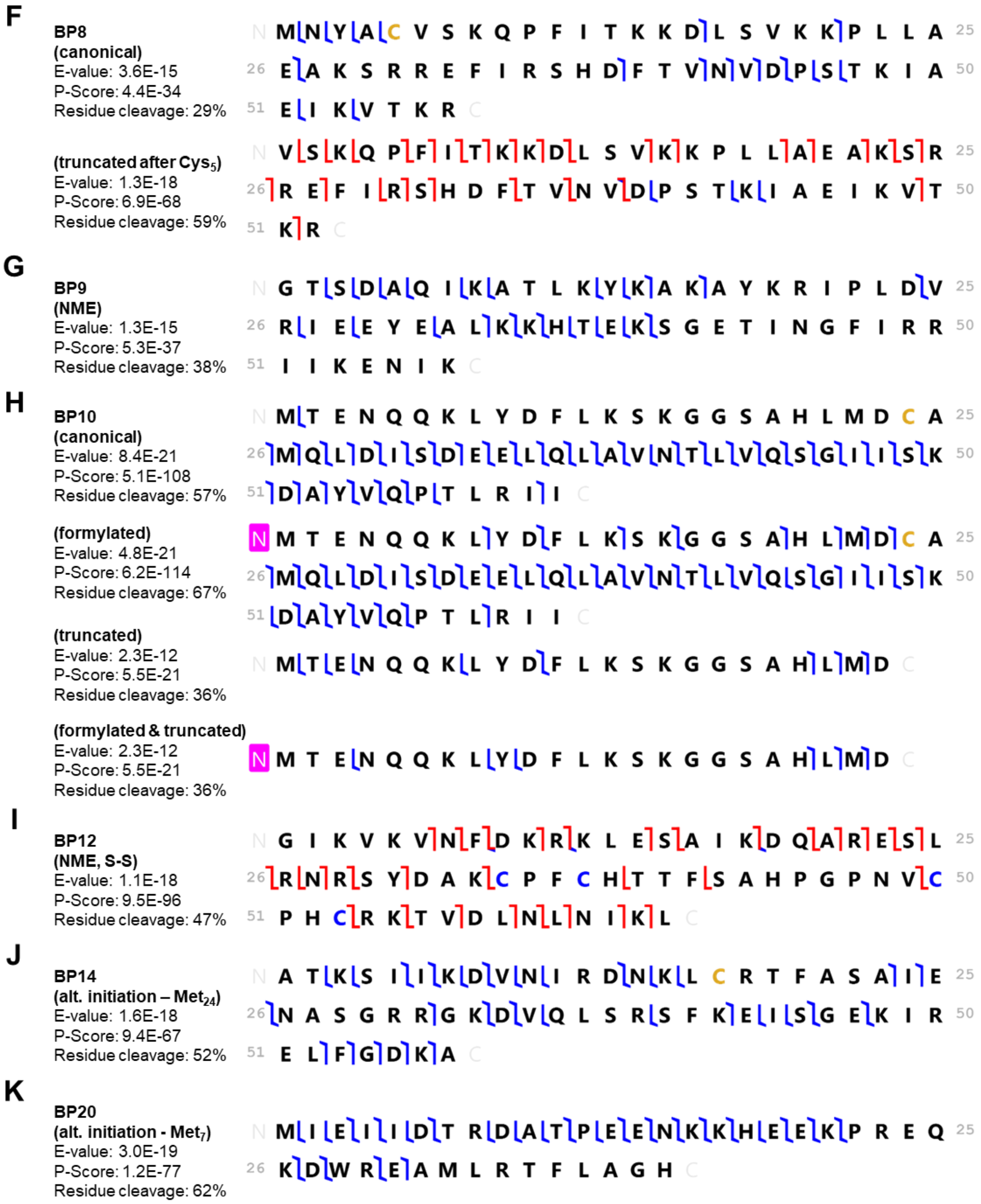

Supplement Figure 3. Examples of a SEP proteoforms identified by top-down proteomics. **(A-K)** displays a selection of 21 proteoforms, from the 52 identified overall, illustrating the diversity of modifications observed. For each proteoform, fragment spectra are displayed, along with the corresponding E-value, P-Score, and residue cleavage information. Abbreviations: *canonical*: full-sequence coverage; NME: N-terminal methionine excision; *alt. initiation*: alternative translation start site; *S-S*: disulfide bridges.

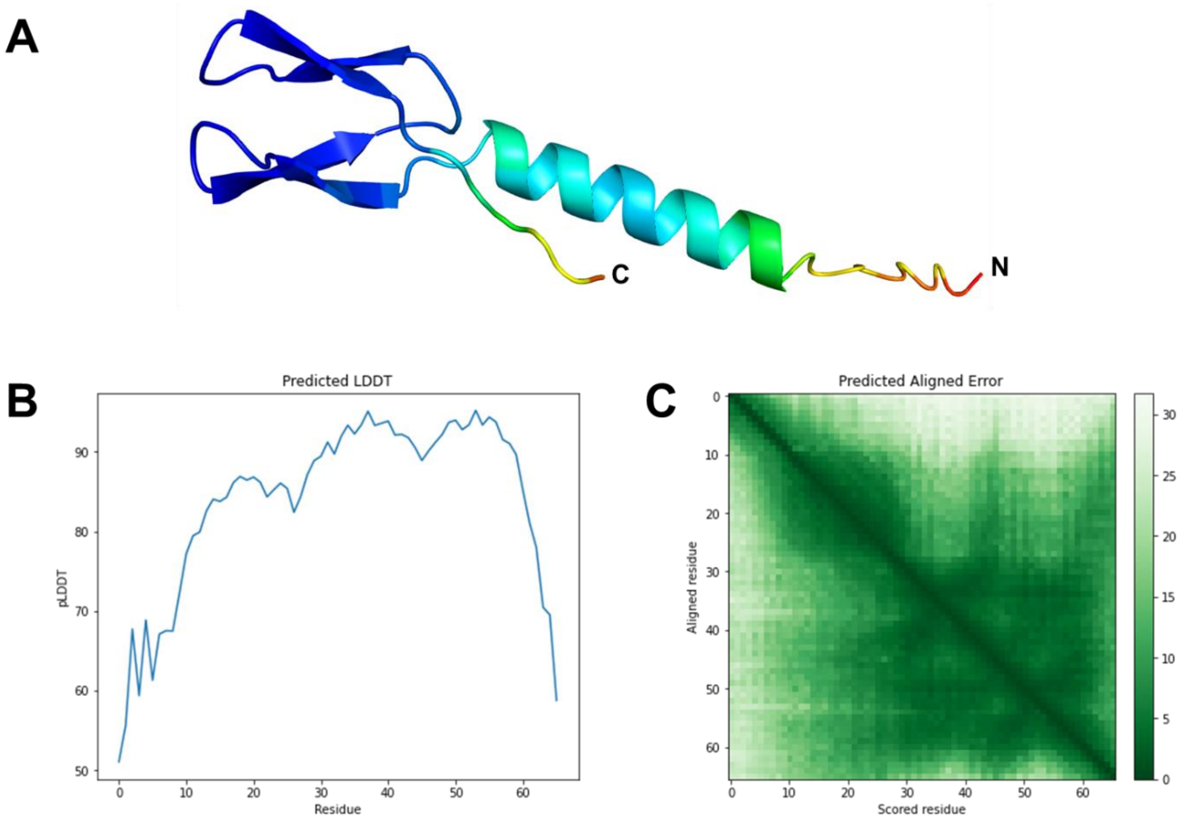

Supplemental Figure 4. Structure prediction of the mature BP12 protein using Alphafold. **(A)** The predicted structure was visualized using PyMol, with the per-residue confidence score (pLDDT) depicted through coloring. **(B)** Plot displaying the predicted LDDT for each residue. **(C)** Predicted position error (in Ångström) for each position.

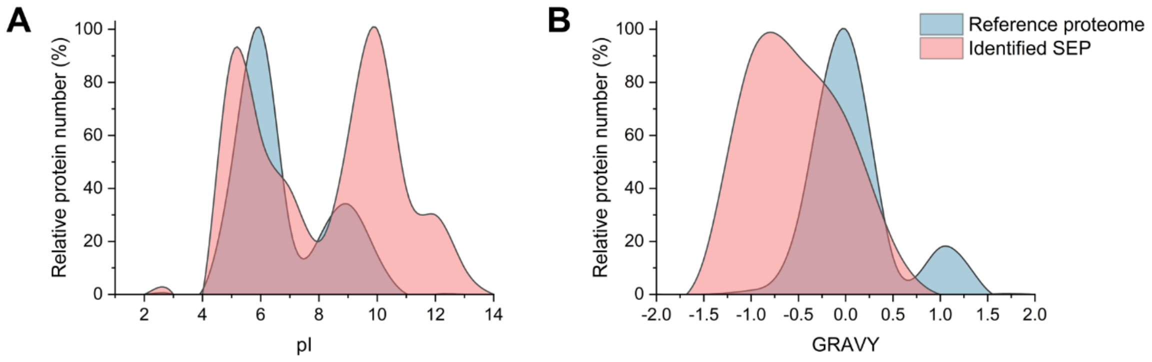

Supplemental Figure 5. Biochemical properties of the identified SEP. Comparison of the **(A)** isoelectric points (pI) and (B) grand average of hydropathy (GRAVY) between the *B. producta* full proteome (blue) and the identified SEP (red).

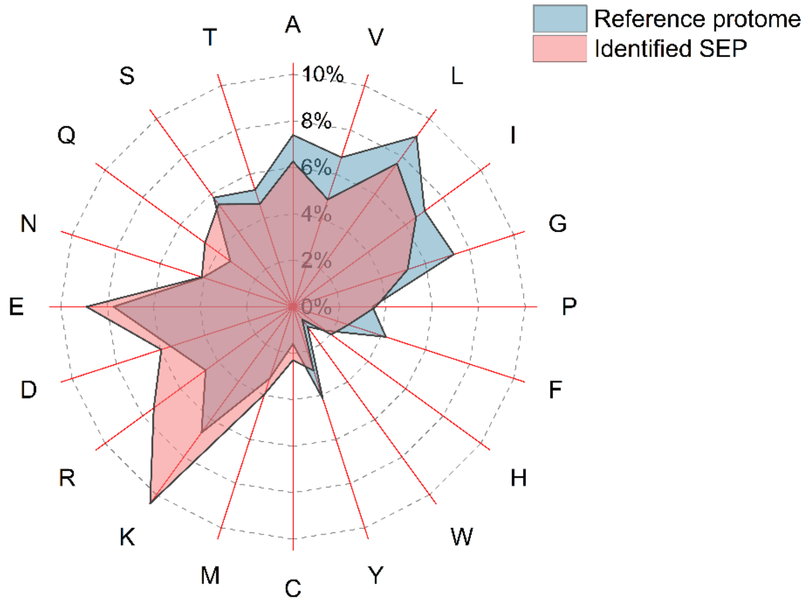

Supplemental Figure 6. Amino acid composition analysis. Comparison of the total amino acid composition of the *B. producta* proteome (blue) and the identified SEP (red).

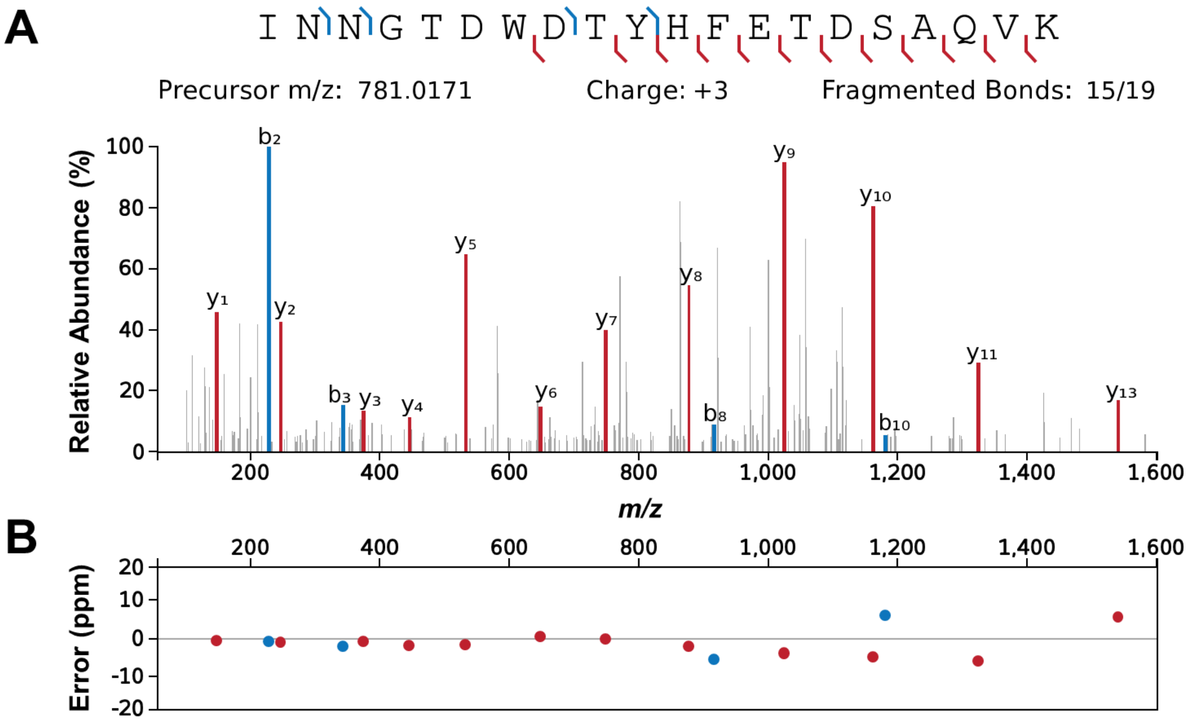

Supplemental Figure 7. Peptide evidence supports the existence for an N-terminal extension of the referenced sequence of the uncharacterized protein (A0A7G5N3A9, E5259_08725). **(A)** The C-terminal peptide INNGTDWDTYHFETDSAQVK was identified with 58 peptide spectrum matches (PSMs). The peptide sequence is annotated with the identified b- and y-ions in blue and red, respectively. Signal deriving from neutral losses (-H_2O, -NH_3, and -CO_2); other ions, except b- and y-ions, are not annotated. **(B)** Dot plots illustrate the mass error in ppm, confirming the correct identification for each observed b and y ion, respectively.
